# Shaping of the 3D genome by the ATPase machine cohesin

**DOI:** 10.1038/s12276-020-00526-2

**Published:** 2020-12-02

**Authors:** Yoori Kim, Hongtao Yu

**Affiliations:** 1 Department of Pharmacology, University of Texas Southwestern Medical Center, Dallas, TX 75390 USA; 2 School of Life Sciences, Westlake University, 310024 Hangzhou, Zhejiang China

**Keywords:** Chromatin structure, Transcriptional regulatory elements

## Abstract

The spatial organization of the genome is critical for fundamental biological processes, including transcription, genome replication, and segregation. Chromatin is compacted and organized with defined patterns and proper dynamics during the cell cycle. Aided by direct visualization and indirect genome reconstruction tools, recent discoveries have advanced our understanding of how interphase chromatin is dynamically folded at the molecular level. Here, we review the current understanding of interphase genome organization with a focus on the major regulator of genome structure, the cohesin complex. We further discuss how cohesin harnesses the energy of ATP hydrolysis to shape the genome by extruding chromatin loops.

## Chromosomes: Seeing the spatial Organization

The cohesin protein complex, an ATP-dependent molecular machine, extrudes DNA into long interwoven loops. The DNA in each cell extends for two meters, but must be compacted into a nucleus of only 10 millionths of a meter in diameter. DNA strands must also be organized so genes are accessible for activation. New imaging techniques now permit visualization of compacted DNA, as reviewed by Hongtao Yu at Westlake University, Hangzhou, China, and Yoori Kim at the University of Texas Southwestern Medical Center, Dallas, USA. After DNA is wrapped around proteins called histones, like beads on a string, cohesin protein complexes attach to the DNA-histone strand and extrude it into loops. Loops, even those from different chromosomes, interweave, permitting efficient grouping of frequently activated regions. This research increases our understanding of the spatial organization of the genome.

## Introduction

The diploid human genome contains 46 chromosomes and 6 billion nucleotides of DNA that, when fully extended, span a length of over 2 m. The genomic DNA has to be folded and confined in the nucleus, which has a dimension of ~10 μm. The compaction of genomic DNA also needs to be dynamic and orderly to allow myriad biochemical reactions that occur on the DNA template, including DNA replication and repair, homologous recombination, and transcription.

The primary level of genome packaging is assembling the DNA into a nucleosome, which consists of 147 bp of DNA wrapping around a histone octamer with two copies each of histone H2A, H2B, H3, and H4. The nucleosomes are separated by linker DNA bound by histone H1. Thus, this 11-nm chromatin fiber has a “beads-on-a-string” structure that compacts DNA approximately 7-fold^1^.

The 11-nm chromatin fiber needs to be further condensed to be packaged in the nucleus. The cohesin complex, an ATP-dependent molecular motor, actively folds chromatin by extruding loops. Chromatin loops are dynamic and preferentially form at certain genomic loci to regulate gene expression and other DNA transactions. In this article, we review our current understanding of the local and global landscapes of interphase chromatin and discuss how cohesin structures chromatin.

## Local folding of interphase chromatin

**Fig. 1:**
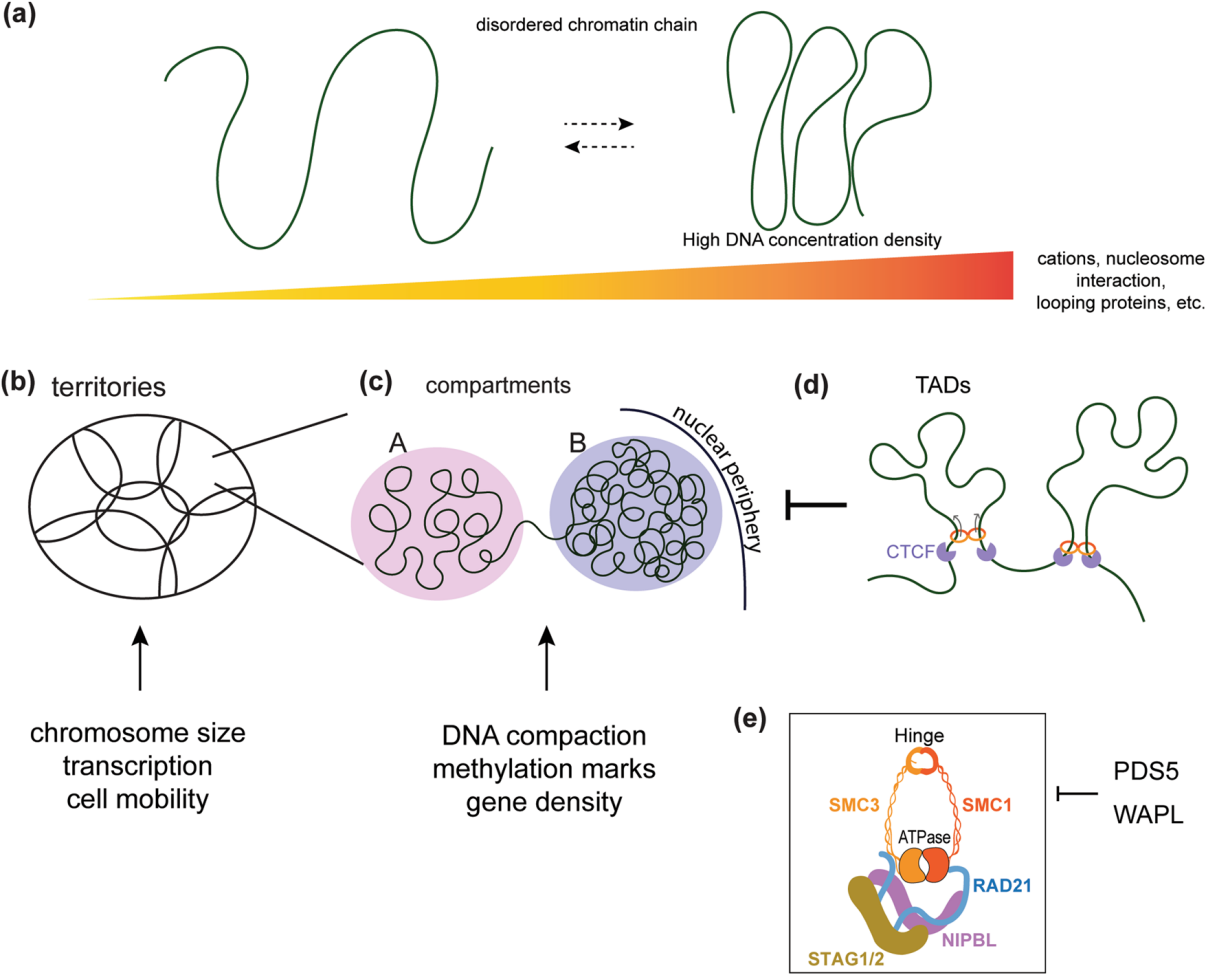
Chromatin organization in the interphase mammalian nucleus. **a** Local view of genome folding. The line indicates 11 nm chromatin fiber. **b**–**d** Global view of interphase chromatin organization. **b** Each chromosome occupies its own territory. **c** Chromatin is spatially divided into A (euchromatin) and B (heterochromatin) compartments. Constitutive heterochromatin is highly compact and positioned at the nuclear periphery. **d** Cohesin and CTCF form topologically associated domains (TADs), which are universal building blocks of chromatin structure. TAD formation counteracts compartment formation. **e** Schematic drawing of cohesin and regulators. Cohesin is a tetrameric complex that consists of SMC1, SMC3, RAD21, and STAG1 or STAG2. NIPBL is required for cohesin loading and for the formation of chromatin loops and TADs. PDS5 and WAPL remove cohesin from chromatin and regulate loop formation.

Until recently, the dominant hypothesis for genome packaging was the hierarchical folding model. This model posits that 11 nm chromatin fiber can fold into a 30 nm fiber, which then folds into thicker 120 and 500 nm fibers and eventually into 1400 nm mitotic chromosomes^2^. However, this model was based on imaging in vitro reconstituted samples, and in vivo evidence was lacking. In fact, recent cryo-electron microscopy (cryo-EM) studies by Eltsov et al. found no evidence for the existence of 30 nm chromatin fibers even in mitotic chromosomes in human cells^3^. Instead, their results suggested chromatin as a highly disordered and interdigitated material. Subsequent studies using cryo-EM and small angle X-ray scattering (SAXS) provided further support for the idea that the mammalian genome is configured as an irregularly folded chromatin fiber and lacks regular periodic structures larger than the 11 nm chromatin fiber^4–6^. The stiffness of the DNA polymer can be assessed by the persistence length. The persistence length of human chromatin in vivo measured using cyclization probability was ~1 kb, which is more consistent with the more flexible 11 nm chromatin fiber than with the stiffer 30 nm fiber^7^. Finally, by developing a labeling method that enhances DNA contrast in electron tomography, Ou et al. showed that human chromatin in situ exists as a disordered 5–24 nm fiber that is packed at different concentration densities in the interphase nuclei and mitotic chromosomes (Fig. 1a)^2^. Taken together, recent findings suggest that chromatin largely exists as flexible 11 nm fibers and do not support the existence of regular, higher-order chromatin assemblies in interphase.

## Global genome configuration in the interphase nucleus

In the interphase nucleus, each decondensed chromosome occupies a discrete nuclear space, termed chromosomal territories^8^. Since the late nineteenth century, the chromosomal territories in interphase nuclei have been visualized by in situ hybridization techniques^8,9^. Their existence has been confirmed by Hi-C, which reveals preferential contacts within the same chromosome. Hi-C is a technique that detects DNA–DNA interactions genome-wide, and matrices of the contact frequencies are routinely displayed as heat maps^10^. The positioning of chromosomal territories is influenced by gene density, chromosome size, and cell geometry (Fig. 1b)^11,12^. Studies using fluorescence in situ hybridization (FISH) and immunostaining have revealed that territorial chromosomes have intermingling regions, which are enriched for transcription markers and exhibit high transcriptional activity^13,14^. In addition, spatial repositioning of chromosomal territories in human cells can occur during the DNA damage response and in aneuploid cells, and altered chromosomal territories are associated with human cancers^12,15–17^. The radial arrangement of chromosomes and interchromosomal communication appear to be critical for diverse cellular responses and for maintaining genome integrity. How cells establish and maintain the spatial positioning of each chromosomal territory is not clear and needs further investigation.

### Compartmentalization

In the 3D nuclear space, interphase chromatin forms two distinct compartments: the A compartment contains gene-rich domains that are concentrated at the center of the nucleus, whereas the B compartment contains gene-poor domains that occupy the exterior nuclear region (Fig. 1c)^18^. Because of their different degrees of compactness, the presence of these two types of compartments was originally observed by differential staining in microscopic imaging. The genome-wide spatial segregation of open and closed chromatin into the A and B compartments can also be observed in the Hi-C map at 1 Mb resolution^19^. These compartments form plaid patterns, suggesting that long-range chromatin interactions occur preferentially within the same compartment.

Numerous studies have established that the gene-dense A compartment is euchromatin, which is characterized by a less compact structure, is highly accessible to DNA-binding proteins, is more active transcriptionally, and exhibits active histone marks, including methylation at H3K4 and, H3K36 and acetylation at H3K27^20,21^. In contrast, compartment B represents transcriptionally repressed heterochromatin, which is highly compacted, contains repetitive DNA elements, and is less susceptible to DNA damage^22–24^. Heterochromatin has been further divided into two types: constitutive heterochromatin near centromeres and telomeres that is enriched for H3K9 methylation and the binding of heterochromatin protein 1 (HP1), and facultative heterochromatin that binds to polycomb group (PcG) proteins and is marked by H3K27 methylation.

Several studies have suggested that the formation of compact heterochromatin is a driver of compartmentalization^25,26^. An attractive model for heterochromatin-driven compartmentalization is liquid–liquid phase separation (LLPS), which can be mediated by multivalent chromatin-binding proteins that spontaneously self-assemble in the presence of nucleic acids^27–29^. This LLPS process results in dense clustering of heterochromatin and its separation from surrounding regions. For example, interactions among chromodomain-containing proteins that bind to H3K9me3 drive LLPS of the H3K9me3 nucleosome arrays^30^, and mutations that disrupt phase separation in vitro also disrupt heterochromatin formation in cells. LLPS has also been suggested to be a mechanism for the establishment of PcG protein-mediated facultative heterochromatin^31,32^.

What are the functional consequences of genome compartmentalization? First, for gene expression, chromatin must be accessible to many sequence-specific DNA-binding proteins that use one-dimensional or three-dimensional diffusion to search for their targets. Gathering gene-dense chromatin at the center of the nuclear space enables such proteins to efficiently navigate and find their targets to execute downstream reactions. Second, genes can be protected from DNA damage by having compact heterochromatin at the nuclear periphery. Third, peripheral chromatin is associated with the lamin network at the nuclear membrane, which is physically connected to the cytoskeleton, and this arrangement enables chromatin remodeling in response to mechanical stimuli^11,33–35^. Thus, compartmentalization plays important roles in organizing and maintaining a functional and responsive genome. How compartmentalization is regulated by chromatin dynamics and cellular responses to stress, such as DNA damage and cell motility, remains an interesting open question.

### Topologically associated domains (TADs)

Higher resolution Hi-C maps revealed the existence of TADs, discrete spatial domains that display high-frequency self-interaction within each unit at 40 kbp resolution^36^. Because TADs are pervasive throughout the genome and are found in all species and cell types, the formation of TADs has been proposed to be a fundamental principle of interphase genome organization^36,37^. Each TAD is separated by distinct boundary regions in which chromatin contacts abruptly end. In mammalian cells, these boundary regions are occupied by the chromatin insulator CTCF and cohesin^38–41^ (Fig. 1d). Each TAD contains multiple chromatin loops, and loops are preferentially anchored at convergent CTCF motifs^42^. TADs have been implicated in cell differentiation and gene regulation^43,44^. The importance of CTCF in genome regulation is also suggested by the fact that various types of human cancers carry mutations at CTCF-binding sites^45,46^.

How do kilobase-sized loop domains of 11 nm chromatin fiber form? The prevailing model is through loop extrusion^47^. One or a pair of loop-extruding factors, such as cohesin, loads on chromatin with two DNA-binding sites and forms a tiny loop. It then drives the processive extrusion of a DNA loop by translocating on DNA, bringing two distant DNA sites into close spatial proximity. Computer simulations and Hi-C data suggested that the loop extrusion model can nicely explain the in vivo genome architecture that features unknotted loops between convergent CTCF sites^7,48^. We will discuss the experimental evidence for cohesin-dependent loop extrusion and the potential mechanisms in later sections.

### Relationships between TADs and compartments

Several lines of evidence suggest that TADs and compartments can compete with each other for formation (Fig. 1d). First, in cells depleted of the cohesin-releasing factor WAPL, increased cohesin on chromatin resulted in the extension of chromatin loops and accumulation of contacts at TAD corners but attenuated contacts in compartments^49^. Conversely, in cells depleted of the cohesin loader NIPBL, the absence of cohesin on chromosomes disrupted TADs but resulted in a finer compartmentalization that is visible as a fragmented plaid pattern in the Hi-C map^50^. Cohesin removal strengthened PcG protein-dependent chromatin domains and resulted in the close proximity of two polycomb domains in embryonic stem (ES) cells^51^. Finally, computer simulation showed that increased processivity of cohesin generated larger TADs while reducing compartmentalization, and deletion of the CTCF boundary affected TADs but not compartments^52^.

A possible function of the TAD-dependent, regulated interruption of compartmentalization is to control the extent of transcriptionally repressive domains and block the formation of an irreversibly silent state of the genome^51^. The mechanism by which cohesin exerts opposing actions on TADs and compartments remains to be defined. One possible way is that the loop extruding activity of cohesin required for TAD formation counteracts the LLPS process, which is a likely driving force of compartmentalization.

## The cohesin complex––an ATPase machine that organizes the genome

Cohesin belongs to the structural maintenance of chromosomes (SMC) family of protein complexes. It was identified in yeast as a key factor required to hold two replicated sister chromatids together and prevent premature sister-chromatid segregation^53^. Cohesin is a tetrameric DNA-binding protein complex comprising SMC1, SMC3, a kleisin subunit RAD21 (Scc1 in yeast), and a HEAT repeat protein STAG1 or STAG2 (Scc3 in yeast)^54,55^. STAG1 and STAG2 are largely redundant in sister-chromatid cohesion but play distinct roles during development^56,57^. The chromatin association of cohesin is dynamically controlled by several regulators. The NIPBL-MAU2 complex mediates cohesin loading, whereas WAPL and PDS5 serve as a cohesin unloading factors (Fig. 1e)^58–60^. Depletion of Wapl or Pds5 in mouse embryonic cells led to clustering of cohesin in axial structures (vermicelli) and caused chromatin condensation in interphase^61^. Cohesin’s chromatin association and dynamics are highly dependent on the cell cycle. In G1 phase, cohesin dynamically associates with chromatin, and upon DNA replication, a pool of cohesin is acetylated and stabilized at the replication fork to mediate sister-chromatid cohesion, which is resolved during mitosis^62,63^.

### ATP-dependent DNA motor activity of cohesin

ChIP-seq data showed that cohesin was enriched at CTCF sites in the genome, which were distinct from its loading sites, and the colocalization of cohesin with NIPBL was increased in ATP-depleted cells^64^. This finding suggested that cohesin loaded on chromatin might undergo ATP-dependent one-dimensional (1D) movement on DNA to reach CTCF sites. Single-molecule experiments showed that human and fission yeast cohesin translocates on DNA by 1D diffusion, which can be blocked by CTCF but not by nucleosomes^65,66^. Such thermal diffusion is a passive random movement that involves the microscopic dissociation and reassociation of a protein complex but is not ATP-dependent DNA motor activity^67–69^.

Recent single-molecule studies have demonstrated that cohesin is indeed a *bona fide* molecular motor that extrudes DNA loops^70,71^. The motor activity of cohesin requires NIPBL. The cohesin-NIPBL complex compacts DNA in an ATP-dependent manner^70^. Once DNA condensation reaches its maximum, only high salt (>300 mM NaCl) treatment can disrupt the compacted cohesin-DNA assemblies; ATP is not required to maintain this compaction^70^. This in vitro finding is consistent with Hi-C results showing that once formed, TADs in human cells no longer require ATP for maintenance^64^. The concentration of cohesin in cells is high, and multiple cohesin molecules are found at each CTCF boundary in vivo^72^. Although the minimal functional unit of loop extrusion can be one or a pair of cohesin-NIPBL molecules, it is likely that multiple cohesin complexes loaded onto a specific regulatory region on chromatin can extrude variable loops until they meet each other and reach the boundary sites. Larger cohesin assemblies might occur on chromatin and help to organize defined chromatin domains and regulate gene expression^73^.

### Symmetric loop extrusion by cohesin complex

Loop extrusion can be achieved in either a symmetric (bidirectional) or an asymmetric (unidirectional) manner. It has been reported that a single condensin complex extrudes a gradually enlarging DNA loop asymmetrically at a fixed anchor point in vitro^74^. To perform asymmetric loop extrusion, the SMC complex needs to use one DNA binding site as the anchor to hold the DNA strand and use other DNA-binding sites to reel in the DNA to enlarge the loop. The Ycg1 HEAT-repeat and Brn1 kleisin subunits of the condensin complex together strap DNA like a “safety belt”, thus serving as the DNA anchor point^75^. The mechanism by which condensin uses ATP hydrolysis to pull in DNA from one direction is not understood.

**Fig. 2:**
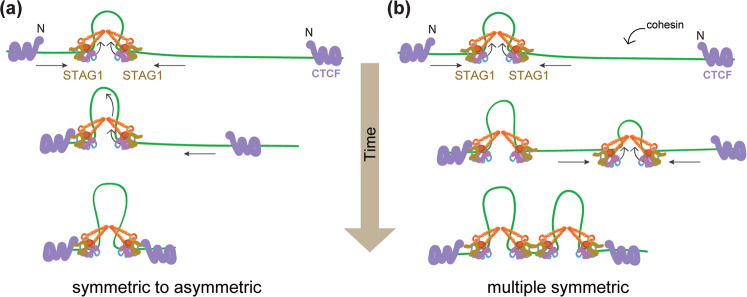
Two models by which symmetric DNA loop extrusion by cohesin is stopped at convergent CTCF sites. **a** A cohesin dimer extrudes a DNA loop bidirectionally towards two CTCF molecules bound at convergent CTCF sites. When STAG1 of one cohesin monomer meets and interacts with the N-terminal region of CTCF, the other cohesin monomer can continue extruding DNA unidirectionally until it also encounters CTCF. **b** Multiple cohesin dimers are loaded onto a genomic region, and each dimer extrudes a DNA loop bidrectionally until it encounters CTCF on one side or another cohesin dimer on the other side.

Unlike condensin, cohesin-NIPBL performs DNA loop extrusion in a symmetric, bidirectional manner^70,71^. Cohesin-NIPBL initially binds at the tip of the “U”-shaped, double tethered DNA and enlarges a DNA loop by moving bidirectionally^70^. These studies clearly demonstrate the loop extrusion activity of cohesin. Whether the loop-extruding cohesin complex is a monomer or a dimer remains to be further clarified^70,71^. A cohesin dimer as the functional unit of loop extrusion can more easily explain the symmetric loop extrusion, as the monomers can reel in DNA from both sides (Fig. 2a, b). Furthermore, a recent study showed that the N-terminal region of CTCF physically interacts with the STAG1/2 subunit of cohesin and is critical for enriching cohesin at CTCF sites^73,76^. A pair of cohesin molecules as the loop extrusion machine is also more consistent with the fact that loop extrusion by cohesin stops at convergent CTCF sites. Because CTCF binds to DNA as a monomer, each CTCF site has one bound CTCF molecule (Fig. 2a, b). The two CTCF molecules at convergent CTCF sites can each interact with the STAG1/2 subunit of one cohesin monomer using the same molecular interface, thus stopping loop extrusion by both. The engagement of one CTCF molecule with one cohesin monomer is expected to stop loop extrusion from one direction, whereas the other cohesin monomer can still perform asymmetric loop extrusion until it encounters another CTCF molecule bound at the second CTCF site (Fig. 2a). Another possibility is that several dimers extrude multiple DNA loops symmetrically until they reach two CTCF sites (Fig. 2b). If cohesin extrudes DNA loops as a monomer, the two CTCF molecules have to interact with cohesin at different, nonoverlapping sites. However, there is currently no evidence for multiple, non-overlapping CTCF-binding sites on cohesin. Future studies are required to clarify the mechanism by which cohesin extrudes DNA loops and how other regulators alter this activity^77,78^.

## Perspectives

Recent advances have revealed key features and principles in the spatial organization of the genome and established cohesin as a major regulator of genome organization. Cohesin organizes the genome by extruding chromatin loops. Regulators of cohesin can impede the loop extrusion activity of cohesin to establish the boundaries of loops and TADs.

Many outstanding questions remain. For example, how does CTCF block the loop extrusion activity of cohesin? How do other cohesin regulators, such as PDS5 and WAPL, counteract cohesin-dependent loop extrusion at CTCF sites? Are there other cellular factors and modifications that impact the processivity and strength of loop extrusion by cohesin? What are the effects of DNA loop extrusion on DNA supercoiling? Addressing these questions will deepen our understanding of the mechanism of action of cohesin, a fascinating ATP-dependent molecular machine that is critical for genome folding and maintenance.
